# Cost-effective length and timing of school closure during an influenza pandemic depend on the severity

**DOI:** 10.1186/1742-4682-11-5

**Published:** 2014-01-21

**Authors:** Hiroshi Nishiura, Keisuke Ejima, Kenji Mizumoto, Shinji Nakaoka, Hisashi Inaba, Seiya Imoto, Rui Yamaguchi, Masaya M Saito

**Affiliations:** 1 Graduate School of Medicine, The University of Tokyo, 7-3-1 Hongo, Bunkyo-ku, Tokyo 1130033, Japan; 2 Department of Mathematical Informatics, Graduate School of Information Science and Technology, The University of Tokyo, 7-3-1 Hongo, Bunkyo-ku, Tokyo 1138656, Japan; 3 Institute of Tropical Medicine and the Global Center of Excellence Program, Nagasaki University, Nagasaki 8528523, Japan; 4 Laboratory for Mathematical Modeling of Immune System, RIKEN Center for Integrative Medical Science Center (IMS-RCAI), 1-7-22, Suehiro-cho, Tsurumi-ku, Yokohama-city, Kanagawa 2300045, Japan; 5 Graduate School of Mathematical Sciences, The University of Tokyo, 3-8-1 Komaba, Meguro-ku, Tokyo 1538914, Japan; 6 Human Genome Center, The Institute of Medical Science, The University of Tokyo, 4-6-1 Shirokanedai, Minato-ku, Tokyo 1088639, Japan; 7 Research and Development Center for Data Assimilation, Institute of Statistical Mathematics, 10-3 Midoricho, Tachikawa, Tokyo 1908562, Japan

## Abstract

**Background:**

There has been a variation in published opinions toward the effectiveness of school closure which is implemented reactively when substantial influenza transmissions are seen at schools. Parameterizing an age-structured epidemic model using published estimates of the pandemic H1N1-2009 and accounting for the cost effectiveness, we examined if the timing and length of school closure could be optimized.

**Methods:**

Age-structured renewal equation was employed to describe the epidemic dynamics of an influenza pandemic. School closure was assumed to take place only once during the course of the pandemic, abruptly reducing child-to-child transmission for a fixed length of time and also influencing the transmission between children and adults. Public health effectiveness was measured by reduction in the cumulative incidence, and cost effectiveness was also examined by calculating the incremental cost effectiveness ratio and adopting a threshold of 1.0 × 10^7^ Japanese Yen/life-year.

**Results:**

School closure at the epidemic peak appeared to yield the largest reduction in the final size, while the time of epidemic peak was shown to depend on the transmissibility. As the length of school closure was extended, we observed larger reduction in the cumulative incidence. Nevertheless, the cost effectiveness analysis showed that the cost of our school closure scenario with the parameters derived from H1N1-2009 was not justifiable. If the risk of death is three times or greater than that of H1N1-2009, the school closure could be regarded as cost effective.

**Conclusions:**

There is no fixed timing and duration of school closure that can be recommended as universal guideline for different types of influenza viruses. The effectiveness of school closure depends on the transmission dynamics of a particular influenza virus strain, especially the virulence (i.e. the infection fatality risk).

## Background

School closure is one of important non-pharmaceutical countermeasures against influenza pandemic [1]. Among various types of school closure, the so-called "proactive closure", i.e., the closure of schools before observing substantial transmissions among school children [1], was conducted in Japan during the early stage of H1N1-2009 pandemic [2], and micro-clade of the viruses that caused the earliest clusters is known to have declined to extinction [3]. Japanese experience demonstrated that the proactive closure as part of concerted effort of containment measure can be very helpful in achieving the local extinction.

However, there has been a variation in published opinions toward another type of closure, "the reactive closure", i.e., the closure of schools when many children, staff or both are experiencing illness, as part of mitigation strategy. Published studies have empirically explored the impact of reactive school closure on an influenza epidemic or pandemic, and some of the studies demonstrated substantial reduction in the rate of transmission among school children during the closure [4-7]. However, others emphasized that the community impact, e.g. reduction in the demand of healthcare service including hospitalization of severe cases, is likely very limited [8-10]. Elucidating the details of school transmission mechanism has been ongoing (see Discussion), and there has been no simple policy (e.g. the timing and duration) to implement the closure in the reactive manner during the course of a pandemic.

If we have a clear quantitative guideline for the reactive closure (e.g. provision of public health conditions at which the closure can be justified and decided), that could greatly benefit public health policymakers. Fundamental insights into the effectiveness of school closure can be gained from a parsimonious mathematical model, exploring possible answers to such key policy questions using simplistic modelling approaches. In the present study, our questions are two-folds. First, we examine when one should close the school during the course of a pandemic. Second, we explore how long the closure should be implemented. Parameterizing the model using published epidemiological estimates of the pandemic H1N1-2009 and accounting for the cost effectiveness of closure, we discuss if the timing and length of school closure could be optimized.

## Methods

### Transmission model

In the present study, the cost effectiveness of school closure is examined using a single-layer epidemic model. Specifically, we consider an age-structured epidemic model that describes the time- and age-dependent transmission dynamics of influenza [11]. Let *j*_a_(*t*) be the incidence (i.e. the number of new infections) of influenza in age-group *a* at calendar time *t*. The renewal process is modelled as

(1) $$j_{a}\left(t\right)=s_{a}\left(t\right)\underset{b}{\sum }\overset{\infty }{\underset{0}{\int }}A_{\text{ab}}\left(s\right)j_{b}\left(t-s\right)\text{ds},$$

where *s*_a_(*t*) is the fraction of susceptible individuals of age-group *a* at time *t*, and *A*_ab_(*s*) stands for the rate of secondary transmission from a single infected individual in age-group *b* to susceptibles in age-group *a* at the infection-age (i.e. the time since infection) *s*, which may be decomposed as

(2) $$A_{\mathrm{ab}}\left(s\right)=R_{\text{ab}}g_{b}\left(s\right),$$

where *R*_ab_ represents the average number of secondary cases in age-group *a* generated by single infected individual in age-group *b*, constituting a single element of the so-called age-dependent "next-generation matrix". *g*_b_(*s*) is the probability density function of the generation time, assumed as dependent on the age-group of primary case. This model can be interpreted as a general representation of the so-called Susceptible-Exposed-Infected-Removed (SEIR) model and its variants in continuous time with a discrete age-structure (e.g. [7,9]). Susceptible individuals are depleted as:

(3) $$s_{a}\left(t\right)=s_{a}\left(0\right)-\frac{\overset{t}{\underset{0}{\int }}j_{a}\left(x\right)\text{dx}}{N_{a}},$$

where *N*_a_ represents the population size of age-group *a*.

### School closure

Let **K** be the age-dependent next-generation matrix, [*R*_ab_] which scales the secondary transmission in (2). In the present study, we consider 3 × 3 matrix, describing within and between group transmissions between/among children, young adults and elderly. That is, we have

(4) $$K=\left(\begin{array}{ccc} R_{11} & R_{12} & R_{13}\\ R_{21} & R_{22} & R_{23}\\ R_{31} & R_{32} & R_{33} \end{array}\right),$$

where subscripts 1, 2 and 3 represent children, young adults and elderly, respectively. The basic reproduction number, *R*_0_, representing the average number of secondary cases produced by a single 'typical’ primary case in a fully susceptible population is computed as the largest eigenvalue of the next-generation matrix (4). During the course of a pandemic, the matrix which describes the age-dependent net reproduction would be scaled by the remaining fraction of susceptibles, *s*_a_(*t*), i.e.,

(5) $$K\left(t\right)=\left(\begin{array}{ccc} s_{1}\left(t\right)R_{11} & s_{1}\left(t\right)R_{12} & s_{1}\left(t\right)R_{13}\\ s_{2}\left(t\right)R_{21} & s_{2}\left(t\right)R_{22} & s_{2}\left(t\right)R_{23}\\ s_{3}\left(t\right)R_{31} & s_{3}\left(t\right)R_{32} & s_{3}\left(t\right)R_{33} \end{array}\right)$$

We assume that **K**(*t*) is decomposed into biological part (e.g. those characterizing susceptibility or infectivity) and contact part (i.e. those associated with contact), i.e.,

(6) $$K\left(t\right)∼\left(\begin{array}{ccc} s_{1}\left(t\right)\alpha_{1} & 0 & 0\\ 0 & s_{2}\left(t\right)\alpha_{2} & 0\\ 0 & 0 & s_{3}\left(t\right)\alpha_{3} \end{array}\right)\;\left(\begin{array}{ccc} M_{11}\Gamma_{1} & M_{12}\Gamma_{2} & M_{13}\Gamma_{3}\\ M_{21}\Gamma_{1} & M_{22}\Gamma_{2} & M_{23}\Gamma_{3}\\ M_{31}\Gamma_{1} & M_{32}\Gamma_{2} & M_{33}\Gamma_{3} \end{array}\right),$$

where *α*_i_ is a relative susceptibility of age group *i*, *M*_ij_ represents the number of contacts that an individual in age group *i* experiences with individuals in age group *j* per unit time, and *Γ*_i_ is the integral of the survival function of infectious period of age group *i* (*Γ*_i_ = 1/*γ*_i_ if the infectious period is exponentially distributed with the mean 1/*γ*_i_ days). Hereafter, the matrix **M** = [*M*_ij_] is referred to as the contact matrix.

In the event of school closure, we assume that the net reproduction matrix is further scaled by a function *ε*(*t*). If the closure influences only the contact among children, we would simplify the issue by rescaling (1,1)-element, i.e.,

(7) $$K'\left(t\right)=\left(\begin{array}{ccc} \varepsilon \left(t\right)s_{1}(t)R_{11} & s_{1}\left(t\right)R_{12} & s_{1}\left(t\right)R_{13}\\ s_{2}\left(t\right)R_{21} & s_{2}\left(t\right)R_{22} & s_{2}\left(t\right)R_{23}\\ s_{3}\left(t\right)R_{31} & s_{3}\left(t\right)R_{32} & s_{3}\left(t\right)R_{33} \end{array}\right),$$

where *ε*(*t*) may be modelled as

(8) $$\varepsilon \left(t\right)=\left\{\begin{array}{l} 1\;\text{for}\;t<t_{0}\;\text{or}\;t\;\geq \;t_{0}\;+\;\tau ,\\ q\;\text{for}\;t_{0}\;\leq \;t<t_{0}\;+\;\tau , \end{array}\right)$$

where *q* represents the relative risk of secondary transmissions during the closure, *t*_0_ represents the starting time of closure, and *τ* stands for the total length of closure. In the existing guideline in Japan, *τ* is suggested to be on the order of 7 days [12] which we regard as our baseline, and examine the possible length up to 50 days. As can be understood from (8), our study considers an epidemic scenario in which the school closure takes place only once. If we additionally account for a compensation of contact with young adults, we assume that the net reproduction matrix is rescaled as

(9) $$K'\left(t\right)=\left(\begin{array}{ccc} \varepsilon \left(t\right)s_{1}(t)R_{11} & \phi \left(t\right)s_{1}(t)R_{12} & s_{1}\left(t\right)R_{13}\\ \phi \left(t\right)s_{2}(t)R_{21} & s_{2}\left(t\right)R_{22} & s_{2}\left(t\right)R_{23}\\ s_{3}\left(t\right)R_{31} & s_{3}\left(t\right)R_{32} & s_{3}\left(t\right)R_{33} \end{array}\right),$$

where *ϕ*(*t*) represents the relative increase in the reproduction number between children and young adults due to compensatory behaviour of children with young adults during the closure. For clarity of modelling and due to shortage of scientific evidence, we ignore the influence of compensation on other elements of the contact matrix (e.g. we assume that the contacts within young adults and between children and elderly are not influenced by closure). Further mathematical details of the compensatory contact are described in Appendix.

### Epidemiological outcomes

Let *z*_a_ represent the final size (i.e. the cumulative incidence) of age group *a*, i.e.,

(10) $$z_{a}=\overset{\infty }{\underset{0}{\int }}j_{a}\left(x\right)\text{dx}-j_{a}\left(0\right)$$

Although mitigation strategy including school closure involves multiple public health objectives (e.g. delaying peak and reducing the height of peak prevalence), the present study focuses on the cumulative risk of infection. Concentrating on this aspect, we explore the possible optimal timing and duration of school closure and examine the cost effectiveness of this countermeasure. Since the average life expectancies are different between age-groups, the loss of life-years, *L*, due to the pandemic is measured by employing age-dependent weighting function, *w*_a_.

(11) $$L=\overset{3}{\underset{a=1}{\sum }}w_{a}z_{a}$$

*w*_a_ is given as the product of the infection fatality risk (IFR; i.e., the risk of death given infection with influenza virus) and life-expectancy of age group *a*, assumed to be 65, 45 and 15 years for children, young adults and elderly, respectively [13]. The age-specific estimates of IFR are extracted from empirical study in Hong Kong, assumed as 1, 10 and 500 deaths per 100,000 infections for children, young adults and elderly, respectively [14]. To measure the effectiveness of school closure, we compare the absolute difference of *L* between two scenarios, i.e. with and without school closure, *L*_1_ and *L*_0_ (i.e., *L*_0_-*L*_1_), yielding the life-years saved by the school closure.

School closure also involves the cost of forced annual leave among parents during the closure. The frequency of such annual leave among Japanese businessmen has been surveyed by Mizumoto et al. [15], showing that up to 16% of daylight time workers were influenced by the school closure and were compelled to take at least an annual leave for half a day. Especially, the parental absenteeism was as high as 26.7% among households with at least one infected child, and this proportion was 10.4% otherwise [15]. Let *c* be the average daily rate of cost induced by annual leave per single young adult. The gross social cost of such parental absenteeism, *G*, is calculated as

(12) $$G=c\tau \left[u_{1}\overset{t_{0}+\tau }{\underset{t_{0}}{\int }}j_{1}\left(x\right)\text{dx}+u_{0}\left(\frac{N_{2}}{2}-\overset{t_{0}+\tau }{\underset{t_{0}}{\int }}j_{1}\left(x\right)\text{dx}\right)\right],$$

where *u*_1_ and *u*_0_ are the proportions of households in which either father or mother has to take annual leave, with or without an infected child in the household, respectively (i.e., *u*_1_ = 0.267 and *u*_0_ = 0.104) and *N*_2_ is the population size of adults. For simplicity, we ignore small fractions of childless couples and unmarried adults among the total young adults. The cost of *u*_1_ is multiplied to the cumulative incidence of children during the closure, ignoring multiple infections in a household (i.e. we ignore brother(s) or sister(s) who are infected at the same time in a household). The population size to multiply *u*_0_ is calculated by subtracting the cumulative incidence from *N*_2_/2, because it is usually the case that either father or mother is absent from work during closure (and one of them continues to work). According to a white paper of an economic study, *c* is given as the product of hourly wage, the average working hours (per day) and the cost to be paid for workers during school closure [16], yielding *c* = 10,019.52 Japanese Yen (approximately 100 US Dollars) per day. Since we consider the short (and realistic) lengths of closure (e.g. 7, 14 or 21 days), we account for only individual impact of closure and ignore other indirect social cost such as the loss of business opportunities due to extended period of closure or stagnation of overall economic activity.

Restricting our scenarios to those reducing epidemic peak by the school closure, we measure the average cost that is required to save a single life year as

(13) $$Y\left(q,\tau ,t_{0},c;R_{\text{ab}},j_{a}\left(0\right)\right)=\frac{G}{L_{0}-L_{1}}$$

It should be noted that the quantity *Y* is theoretically equivalent to the incremental cost effectiveness ratio (ICER) in the cost effectiveness analysis (CEA) studies. We aim to identify reasonable combination of *τ* and *t*_0_ that minimizes our objective function *Y*. In the United States and United Kingdom, the acceptable threshold of ICER tends to lie around 100,000 US dollars and 30,000 British Pound per life year, respectively. Accordingly, we assume that the corresponding threshold lies in the range from 5.0 × 10^6^ to 1.0 × 10^7^ Japanese Yen (and draw a line for the latter threshold in all associated figures).

### Parameter setting

We consider an epidemic in a population of 1 million. The size of each age group is assumed as proportional to age-specific population sizes of those aged from 0–19, 20–59 and 60 years and over for the entire Japan, i.e., (*N*_1_, *N*_2_, *N*_3_) = (177207, 500957, 321836). The parameters of contact matrix are derived from previous study with an identical age categorization [17] which essentially assumed that the age-specific contact pattern in Japan is not different from that in England [18]. In our scenario, an epidemic takes place with an introduction of single infected child *j*_1_(0) = 1 (while *j*_2_(0) = *j*_3_(0) = 0), and all residents are assumed as initially susceptible, i.e., (*s*_1_(0), *s*_2_(0), *s*_3_(0)) = (1,1,1). Nevertheless, their relative susceptibility per contact (e.g. the probability of successful transmission per contact) depends on age, i.e., (*α*_1_, *α*_2_, *α*_3_) = (1.000,0.370,0.059) as empirical evidence suggests [11,19]. Mean generation times of secondary transmission caused by children and young adults or elderly are assumed to be 2.2 days and 2.7 days, respectively [20], and we assume that the generation time follows an exponential distribution for mathematical convenience (so that the model (1) can also be written as ordinary differential equations using the next-generation matrix parameterized by (6)). The basic reproduction number, *R*_0_, is used for scaling the next-generation matrix and is assumed to be 1.4 [21,22].

With respect to the protective effect of intervention, the relative reduction in the reproduction number during school closure (hereafter referred to as the "efficacy" of school closure) has been empirically estimated in limited number of settings [4,5,9,23]. As our baseline, we assume that there is a 70% decline in child-to-child transmission during the closure and thus *q* = 0.3 [5]. In a Japanese survey [15], no apparent increase in the frequency of child-to-adult contact was observed during the closure, and we set the proportion of child contacts compensated (*π*; see Appendix) as 0 at the baseline and then vary it from 0 to 0.5 (where 0.5 means that 50% of intervened within-child contacts are alternatively made with young adults).

The above-mentioned parameters correspond to empirically measured results from pandemic H1N1-2009 which is known to have been very mild [24]. Thus, we also measure the sensitivity of ICER to different levels of transmission potential and risks of death, varying *R*_0_ from 1.2 to 1.8 and elevating the relative risk of death from 1 to 100 (using H1N1-2009 as the reference).

## Results

### Epidemic dynamics and school closure

**Figure 1:**
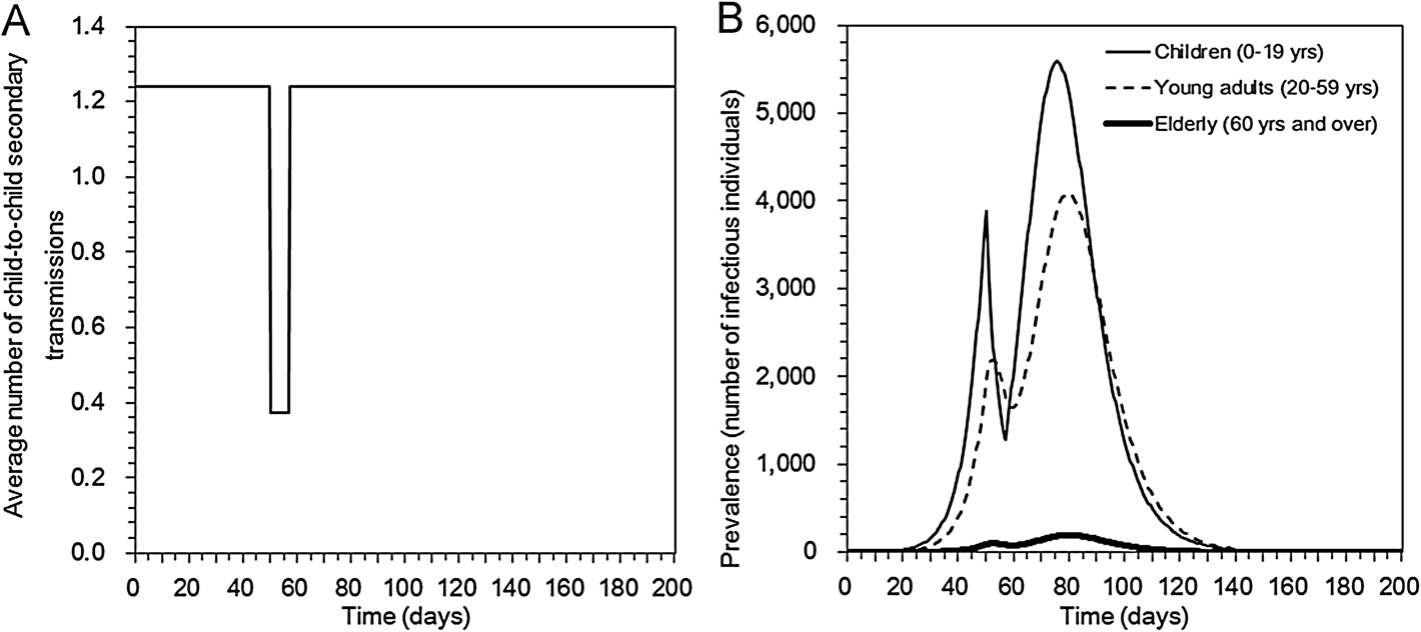
**A scenario of school closure during the course of an influenza pandemic.** School closure is implemented for 7 days from Day 50. The basic reproduction number is set at 1.4. Panel **A**. The average number of child-to-child secondary transmissions in the absence of the depletion of susceptibles. There is an abrupt 70% decline in the child-to-child secondary transmissions, while no compensatory contact with adults is assumed at the baseline. **B**. Age-dependent prevalence (i.e. the age-specific number of infectious individuals) as a function of calendar time.

Figure 1 illustrates a single scenario of influenza pandemic with school closure. At a specified epidemic day, we assumed that school closure is initiated, abruptly reducing the number of child-to-child secondary transmissions by a constant factor for a fixed length of time, and the transmission potential recovers when the school is reopened (Figure 1A). A sharp decline in the child incidence was observed, and it also influenced the transmission dynamics of adults and elderly. In the absence of school closure, the cumulative incidence of children, young adults and elderly were 103242 (58.2%), 66156 (13.2%) and 3142 (0.9%), respectively, yielding the final size of 17.3% for the entire population. These figures were in line with the result from seroepidemiological survey of H1N1-2009 in Japan [25]. The highest incidence was observed at Day 61. When the school closure is implemented at Day 50 for 7 days, the cumulative incidence of children, young adults and elderly were 95795 (54.1%), 61967 (12.4%) and 2930 (0.9%), respectively, yielding the final size of 16.1% for the entire population. The highest incidence was observed at Day 75.

**Figure 2:**
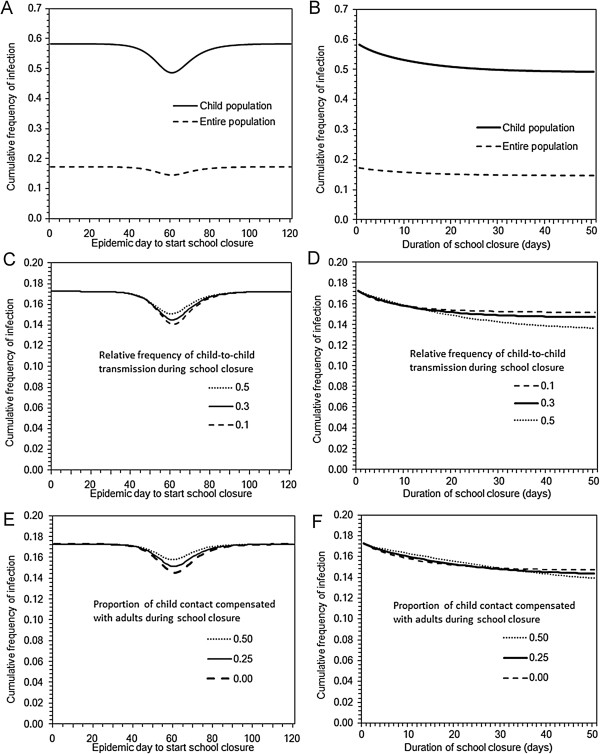
**Cumulative incidence of pandemic with different timing and lengths of school closure.** Panels **A**, **C** and **E** examine the sensitivity of the cumulative incidence (i.e. final size) to different timing of school closure. Length of school closure in these panels is set at 7 days. Similarly, Panels **B**, **D** and **F** explore the sensitivity of the cumulative incidence to different lengths of school closure. Timing of school closure in these panels is set at Day 50. The basic reproduction number is set at 1.4. Panels **A** and **B** compare the final size among children to that of the entire population. Panels **C** and **D** vary the efficacy of school closure (i.e. the relative reduction in the child-to-child secondary transmissions) from 50% to 90%. No compensatory contact with adults is assumed in these panels. Panels **E** and **F** vary the proportion of child contact compensated with young adults, assuming that the compensation occurs for 0 to 50% of intervened within-child contacts. In these panels, the efficacy of school closure is set at 70%.

Figure 2 shows the sensitivity of the cumulative incidence to variable timing and lengths of school closure. The cumulative incidence was minimized if the closure was started at Day 61. Namely, the largest reduction of final size was observed by implementing the closure at the peak of the epidemic (Figure 2A). Moreover, as the length of school closure was extended, the cumulative incidence was decreased (Figure 2B). Based on our univariate sensitivity analysis, these findings were only marginally influenced by different efficacy of school closure (i.e. variations in the relative reduction in the child-to-child secondary transmissions had little impact on the cumulative incidence) (Figures 2C and 2D) and variable compensatory behaviour of children with young adults (Figures 2E and 2F).

### Cost effectiveness analysis of school closure

**Figure 3:**
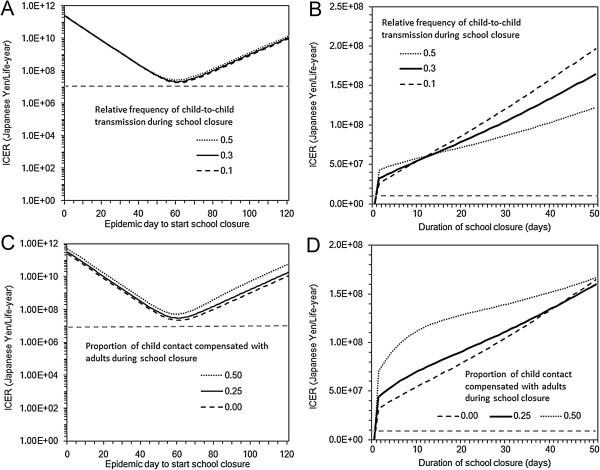
**Cost effectiveness of school closure with different timing and lengths.** Incremental cost effectiveness ratio (ICER), expressed as Japanese Yen per single life-year saved, is computed. Horizontal dashed grey line represents the threshold value of ICER, 1.0 × 10^7^ Yen/life-year, below which one may regard the intervention as cost-effective. Panels **A** and **C** examine the sensitivity of ICER to different timing of school closure. Length of school closure in these panels is set at 7 days. Similarly, Panels **B** and **D** explore the sensitivity of ICER to different lengths of school closure. Timing of school closure in these panels is set at Day 50. The basic reproduction number is set at 1.4. Panels **A** and **B** vary the efficacy of school closure (i.e. the relative reduction in the child-to-child secondary transmissions) from 50% to 90%. No compensatory contact with adults is assumed in these panels. Panels **C** and **D** vary the proportion of child contact compensated with young adults, assuming that the compensation occurs for 0 to 50% of intervened within-child contacts. In these panels, the efficacy of school closure is set at 70%.

Figure 3 shows ICER values with various timing and lengths of school closure. As there was an optimal timing to minimize the final size in Figure 2, the ICER also took the minimum value when the closure is implemented at the epidemic peak (Figures 3A and 3C). Nevertheless, it is noteworthy that the ICER remained to be above the acceptable threshold for all the assumed parameter space for H1N1-2009 (even when ICER took the minimum value). As for the length of closure, the ICER appeared to be a monotonically increasing function of the length of closure. For both the timing and length, the variations in the efficacy of closure and proportion of contacts compensated had only marginal impact on ICER.

**Figure 4:**
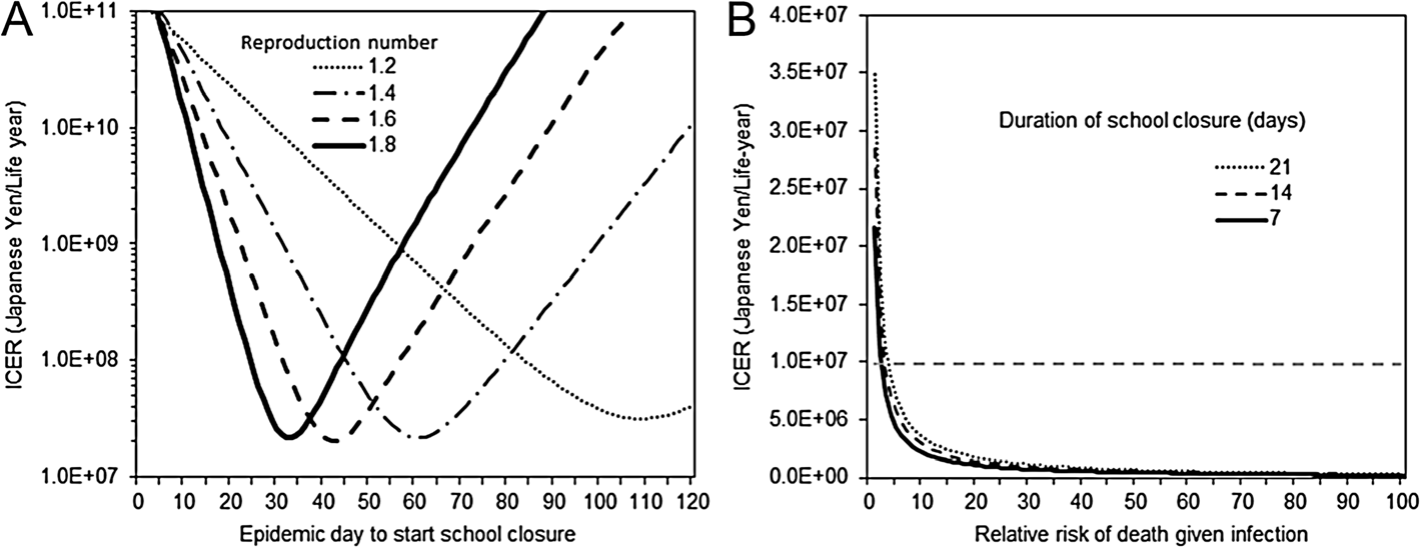
**Sensitivity of the cost effectiveness of school closure to the severity of influenza pandemic.** Incremental cost effectiveness ratio (ICER), expressed as Japanese Yen per single life-year saved, is computed. Panel **A** examines ICER as a function of the reproduction number (ranging from 1.2 to 1.8) and the timing of school closure. Length of school closure in these panels is set at 7 days. It should be noted that the horizontal axis is at the ICER of 1.0 × 10^7^ Yen/life-year. Panel **B** shows ICER as a function of the risk of death relative to assumed baseline of H1N1-2009 pandemic and the length of school closure. The relative risk of death of assumed pandemic is expressed as multiplier to the infection fatality risk of H1N1-2009 (e.g. if the relative risk is 50, the assumed pandemic is 50 times more likely lethal upon infection). The basic reproduction number and the timing of school closure in these panels are set at 1.4 and Day 60, respectively. Horizontal dashed grey line represents the threshold value of ICER, 1.0 × 10^7^ Yen/life-year, below which one may regard the intervention as cost-effective.

Figure 4 examines the ICER by varying two additional epidemiological variables, i.e., the basic reproduction number (Figure 4A) and the relative risk of death given infection (Figure 4B). The timing of epidemic peak varied with *R*_0_, and thus, the time for ICER to take the minimum value also greatly varied with *R*_0_. Greater *R*_0_ yielded the minimum ICER value at earlier epidemic time. Nevertheless, again all the ICER values were above the acceptable threshold in Figure 4A. When the infection fatality risk was proportionally magnified, we found that the ICER fall below acceptable threshold. That is, when the risk of death was three times or greater than that of H1N1-2009, all scenarios that we examined appeared to be cost effective.

## Discussion

The present study examined the public health effectiveness and cost effectiveness of school closure which was assumed to be implemented only once during the course of a pandemic. The model was parameterized with reference to empirical estimates of the pandemic H1N1-2009. School closure at the epidemic peak appeared to minimize the cumulative incidence, but the time of epidemic peak was shown to depend on *R*_0_. As the duration of school closure was extended, we observed a larger reduction in the cumulative incidence. Strikingly, the cost effectiveness analysis showed that our school closure scenario with parameters derived from the pandemic H1N1-2009 was not cost effective. Nevertheless, if the virulence is three times or greater than that of H1N1-2009, the cost of closure could be justified. These findings were not very sensitive to the efficacy of school closure and compensatory contact behaviour among children.

There are three important learning points from the present study. First, we have shown that there is no fixed timing and duration of school closure that can be recommended as universal for different types of influenza viruses. It is natural that the effectiveness of school closure depends on the underlying transmission dynamics, and the absence of simple universal guideline should be explained and communicated to non-experts. In fact, the dependence of the effectiveness of school closure on the transmission dynamics could explain variations in published opinions toward the epidemiological performance of the school closure as an option of mitigation strategy. Second, school closure during the pandemic H1N1-2009 appeared not to be cost effective even when the ICER took the minimum value. It implies that the cost-effective intervention against mild pandemic strain such as H1N1-2009 is different from that of virulent strains. This echoes the finding by Halder et al. [26,27] based on a simulation approach. Third, if a particular strain is virulent, school closure could be cost effective. Namely, given that the virulent strain widely spreads in the community, yielding high disease burden, school closure intervention, the cost of which is regarded as small for the high disease burden, should be implemented to reduce the disaster size as much as possible. In addition, since the equation (12) involves the wage of parents, it is important to remember that the cost-effectiveness may also depend on an economic standard of a country (e.g. school closure of pandemic H1N1-2009 may even be justified in a country with much smaller salary than that of Japan).

As a policy implication at minimum, one should remember that optimal school closure depends on the severity of pandemic, characterized by the transmissibility and virulence. Especially, the school closure is likely cost effective for virulent influenza strains. One should also know that the cost-effective interventions of a particular influenza strain are different from those for other strains. In addition, rather than industrialized countries, it may be easier to justify the cost of school closure in developing countries where the parental impact is likely smaller.

Three technical limitations should be noted. First, our model assumes that the transmission from child to child is homogeneous. More rigorous network model has shown that such random mixing assumption could overestimate the effectiveness of school closure [28]. Second, the impact of school closure on social mixing patterns should ideally be based on more realistically socially structured (layered) modelling approach [29], perhaps classifying transmissions into those occurring in households, schools and community [30]. Third, more precise features of child contact, including weekend contact and the impact of illness on the contact, are recently shown to have a substantial impact on the effectiveness of intervention [31,32].

Unfortunately, school closure during H1N1-2009 may not be fully justified when it comes to the cost. Nevertheless, the cost effectiveness should be regarded as merely a single aspect of the impact of this intervention to help policymaking. Perhaps, rather than focusing more on the cost, one should carefully reconsider public health objectives of this intervention, e.g. delaying epidemic peak, reducing the height of peak prevalence, or reducing the overall epidemic size, and decide what we would expect from this intervention more in detail. Expecting the effectiveness in all these aspects may not be feasible [33]. Despite the presence of numerous tasks to guide school closure in the next pandemics, our study has at least shown that one can examine the potential performance of school closure using the proposed simplistic modelling approach.

## Conclusions

The present study examined the public health effectiveness and cost effectiveness of school closure. The effectiveness of school closure depends on the transmission dynamics of a particular influenza virus strain, especially the virulence. School closure in our scenario with parameters derived from the pandemic H1N1-2009 appeared not to be cost effective. There is no fixed timing and duration of school closure that can be recommended as universal guideline for different types of influenza viruses.

## Appendix

Here we describe the mechanism of compensatory contact of children during school closure. Let M^*^ be a symmetric matrix that represents the rate of total contacts made by each age-group. That is, supposing that the population sizes of children, young adults and elderly are expressed as (*N*_1_, *N*_2_, *N*_3_), we have

(A1) $$M^{*}=M\left(\begin{array}{ccc} N_{1} & 0 & 0\\ 0 & N_{2} & 0\\ 0 & 0 & N_{3} \end{array}\right)$$

Sum of elements in a single row *i* or single column *i* represents the total number of contacts (per day) made by all of those in age-group *i*. We assume that the sum of each column is decomposed as the product of average contact per person *k*_i_ and the population size *N*_i_, so that the sum can be rewritten as *k*_i_*N*_i_. In the presence of compensatory behaviour, we assume that the total number of contact, *k*_i_*N*_i_, made by age-group *i* is partially maintained even during the school closure.

Let *p*_1_ and *p*_2_ be the proportion of child contacts spent for children and young adults, then the first row (or first column) of M^*^ should read (*p*_1_*k*_1_*N*_1_, *p*_2_*k*_1_*N*_1_, (1-p_1_-p_2_)*k*_1_*N*_1_). Similarly, let *p*_3_ be the proportion of young adult contacts for other young adults or elderly that are spent for young adults. We get

(A2) $$M^{*}=\left(\begin{array}{ccc} p_{1}k_{1}N_{1} & p_{2}k_{1}N_{1} & \left(1-p_{1}-p_{2}\right)k_{1}N_{1}\\ p_{2}k_{1}N_{1} & p_{3}\left(k_{2}N_{2}-p_{2}k_{1}N_{1}\right) & \left(1-p_{3}\right)(k_{2}N_{2}-p_{2}k_{1}N_{1})\\ \left(1-p_{1}-p_{2}\right)k_{1}N_{1} & \left(1-p_{3}\right)(k_{2}N_{2}-p_{2}k_{1}N_{1}) & k_{3}N_{3}-\left(1-p_{1}-p_{2}\right)k_{1}N_{1}-(1-p_{3})(k_{2}N_{2}-p_{2}k_{1}N_{1}) \end{array}\right)$$

Thus, the contact matrix **M** is given by

(A3) $$M=M^{*}\left(\begin{array}{ccc} \frac{1}{N_{1}} & 0 & 0\\ 0 & \frac{1}{N_{2}} & 0\\ 0 & 0 & \frac{1}{N_{3}} \end{array}\right)$$

In the event of school closure, the contact rate among school children is expected to decrease. If the impact of school closure were seen only among children, we would have the following contact matrix during the closure, M*, as

(A4) $$M^{'}=\left(\begin{array}{ccc} \varepsilon \left(t\right)p_{1}k_{1} & \frac{p_{2}k_{1}N_{1}}{N_{2}} & \frac{\left(1-p_{1}-p_{2}\right)k_{1}N_{1}}{N_{3}}\\ p_{2}k_{1} & \frac{p_{3}\left(k_{2}N_{2}-p_{2}k_{1}N_{1}\right)}{N_{2}} & \frac{\left(1-p_{3}\right)\left(k_{2}N_{2}-p_{2}k_{1}N_{1}\right)}{N_{3}}\\ \left(1-p_{1}-p_{2}\right)k_{1} & \frac{\left(1-p_{3}\right)\left(k_{2}N_{2}-p_{2}k_{1}N_{1}\right)}{N_{2}} & k_{3}-\frac{\left(1-p_{1}-p_{2}\right)k_{1}N_{1}+\left(1-p_{3}\right)\left(k_{2}N_{2}-p_{2}k_{1}N_{1}\right)}{N_{3}} \end{array}\right),$$

where, as in the main text, *ε*(t) may be modelled as

(A5) $$\varepsilon \left(t\right)=\left\{\begin{array}{l} 1\;\text{for}\;t<t_{0}\;\text{or}\;t\;\geq \;t_{0}\;+\;\tau ,\\ q\;\text{for}\;t_{0}\;\leq \;t<t_{0}\;+\;\tau . \end{array}\right)$$

Nevertheless, school closure could, in theory, influence the contacts with other age-groups too. That is, school children are likely to stay in the home during the closure and the contact rate between children and adults may be increased in an indirect manner, e.g., parents may have to spend longer time with children than usual. If the proportion *π* of reduced child-to-child contacts is maintained and compensated by child-to-young adult contacts, the contact matrix during the closure may read

(A6) $$M^{'}=\left(\begin{array}{ccc} qp_{1}k_{1} & \frac{\left(1-q\right)\pi p_{1}k_{1}N_{1}+p_{2}k_{1}N_{1}}{N_{2}} & \frac{\left(1-p_{1}-p_{2}\right)k_{1}N_{1}}{N_{3}}\\ \left(1-q\right)\pi p_{1}k_{1}+p_{2}k_{1} & \frac{p_{3}\left(k_{2}N_{2}-p_{2}k_{1}N_{1}\right)}{N_{2}} & \frac{\left(1-p_{3}\right)\left(k_{2}N_{2}-p_{2}k_{1}N_{1}\right)}{N_{3}}\\ \left(1-p_{1}-p_{2}\right)k_{1} & \frac{\left(1-p_{3}\right)\left(k_{2}N_{2}-p_{2}k_{1}N_{1}\right)}{N_{2}} & k_{3}-\frac{\left(1-p_{1}-p_{2}\right)k_{1}N_{1}+\left(1-p_{3}\right)\left(k_{2}N_{2}-p_{2}k_{1}N_{1}\right)}{N_{3}} \end{array}\right),$$

It should be noted that the increase in child contact with elderly is ignored, assuming that mostly parents, not elderly, have to take care of children during the school closure [15]. It should also be noted that other contacts were assumed not to have been influenced by the compensation, although, in theory, the maintenance of contacts should influence all other elements (so that the total number of contacts per day remain constant for all age-groups); we ignore this mathematical issue for simplicity.

## Competing interests

The authors declare that they have no competing interests.

## Authors’ contributions

This study was conceived, structured and agreed by all authors during a Study Group session including the conception of study, methodological development, implementation and interpretation. HN implemented computational analyses, interpreted the results and drafted the manuscript. KE and SN revised the model. KM tracked published information to parameterize the model. HI, SI, RY and SM discussed and gave comments on the earlier version of the manuscript. All authors read and approved the final manuscript.
